# A Homœopathic Hospital in Italy

**Published:** 1889-06

**Authors:** 


### A HOMŒOPATHIC HOSPITAL IN ITALY.

The April number of the *Rivista Omiopatica*, a journal published in Rome, under the direction of Dr. G. Pompili, contains an article under the above heading, so interesting that we specially call the attention of our readers to it.

A benevolent and generous citizen of Verona, Joseph Camploy, died on the 12th of last February in Venice, at the advanced age of ninety-five years, leaving a will, in which he bequeathed the whole of his property to the city of his birth, for the purpose of founding a hospital, to be called "The Hahnemannian Hospital, Camploy."

According to the terms of the will, only Hahnemannian Homœopathy is to be practiced in it.

Dr. G. Pompili, of Rome, is appointed medical director for life. He will name his successor, who must be a Hahnemannian, and indorsed by the municipality of Verona as such.

This second director must in turn name his successor, and thus it is hoped the Hahnemannian character of the appointments will be maintained.

A resident-physician is provided for at a salary of 150 lire a month (less than thirty dollars).

The will concludes: "In giving such an institution to Verona, I hope and feel that it will bring a great benefit to my countrymen, as Hahnemann's method is the only method of curing simply, surely, and perfectly."

Dr. Mattoli, who contributes this article to the *Rivista*, adds the following comment:

"We here perceive that our good friend, Camploy, does not understand any deviation from the strict teachings of Hahnemann; he recognizes no physicians who mix allopathy and Homœopathy, and calls all such mongrels." [Eds.]

